# Supplementary material for: Physiologically Based Pharmacokinetic Modeling of Antibiotics in Children: Perspectives on Model-Informed Precision Dosing
Source: Antibiotics (Basel). 2025 May 24;14(6):541. doi: 10.3390/antibiotics14060541 (PMC12189772; doi:10.3390/antibiotics14060541)
Supplement: Supplementary file 1 [file antibiotics-14-00541-s001.zip › Table S2_Articles initially retrieved.pdf]

Table S1 Articles initially retrieved 2016/1-2024/11

No.	Title	First Author	Journal/Book	Publication Year	DOI
1	Prediction of tissue exposures of polymyxin-B, amikacin and sulbactam using physiologically-based pharmacokinetic modeling	Wu M	Front Microbiol	2024	10.3389/fmicb.2024.1435906
2	Physiological modeling of the metaverse of the Mycobacterium tuberculosis beta-CA inhibition mechanism	Giovannuzzi S	Comput Biol Med	2024	10.1016/j.compbiomed.2024.109029
3	PBPK Modeling of Entrectinib and Its Active Metabolite to Derive Dose Adjustments in Pediatric Populations Co-Administered with CYP3A4 Inhibitors	Umehara K	Clin Pharmacol Ther	2024	10.1002/cpt.3386
4	Age-Dependent Abundance of CYP450 Enzymes Involved in Metronidazole Metabolism: Application to Pediatric PBPK Modeling	Parvez MM	Clin Pharmacol Ther	2024	10.1002/cpt.3354
5	Skin pharmacokinetics of miltefosine in the treatment of post-kala-azar dermal leishmaniasis in South Asia	Palić S	J Antimicrob Chemother	2024	doi: 10.1093/jac/dkae129
6	Population pharmacokinetic analysis for dose regimen optimization of vancomycin in Southern Chinese children	Shen X	CPT Pharmacometrics Syst Pharmacol	2024	10.1002/psp4.13151
7	Physiologically-Based Pharmacokinetic Modeling and Dosing Optimization of Cefotaxime in Preterm and Term Neonates	Li Q	J Pharm Sci	2024	10.1016/j.xphs.2024.03.002
8	Maximum likelihood estimation of renal transporter ontogeny profiles for pediatric PBPK modeling	Hunt JP	CPT Pharmacometrics Syst Pharmacol	2024	10.1002/psp4.13102
9	Development and application of neonatal physiology-based pharmacokinetic models of amikacin and fosfomycin to assess pharmacodynamic target attainment	Darlow CA	CPT Pharmacometrics Syst Pharmacol	2024	10.1002/psp4.13097
10	Meropenem extraction by ex vivo extracorporeal life support circuits	Honeycutt CC	J Extra Corpor Technol	2023	10.1051/ject/2023035
11	Preliminary evidence for sustained efficacy of CFTR modulator therapy with concomitant rifabutin administration	Hong E	J Cyst Fibros	2024	10.1016/j.jcf.2023.11.015
12	Development of a Generic Fetal Physiologically Based Pharmacokinetic Model and Prediction of Human Maternal and Fetal Organ Concentrations of Cefuroxime	Liu XI	Clin Pharmacokinet	2024	10.1007/s40262-023-01323-6
13	Formulation development, in vivo bioequivalence and pediatric PBPK modeling studies of taste-masked ciprofloxacin chewable tablets	Usmani MT	Sci Rep	2023	10.1038/s41598-023-43423-0
14	Vancomycin population pharmacokinetics analysis in Chinese paediatric patients with varying degrees of renal function and ages: development of new practical dosing recommendations	Chen J	J Antimicrob Chemother	2023	10.1093/jac/dkad202
15	Development of a Minimalistic Physiologically Based Pharmacokinetic (mPBPK) Model for the Preclinical Development of Spectinamide Antibiotics	Parmar KR	Pharmaceutics	2023	10.3390/pharmaceutics15061759
16	Physiologically-Based Pharmacokinetic Modelling to Predict the Pharmacokinetics and Pharmacodynamics of Linezolid in Adults and Children with Tuberculous Meningitis	Litjens CHC	Antibiotics (Basel)	2023	10.3390/antibiotics12040702
17	Develop adult extrapolation to pediatrics and pediatric dose optimization based on the physiological pharmacokinetic model of azithromycin	Liang L	Biopharm Drug Dispos	2023	10.1002/bdd.2352
18	Physiologically based pharmacokinetic modelling to inform combination dosing regimens of ceftaroline and daptomycin in special populations	Martins FS	Br J Clin Pharmacol	2023	10.1111/bcp.15731
19	Pan-Genomics of Escherichia albertii for Antibiotic Resistance Profiling in Different Genome Fractions and Natural Product Mediated Intervention: In Silico Approach	Jalal K	Life (Basel)	2023	10.3390/life13020541
20	Predictive Performance of Physiologically Based Pharmacokinetic Modelling of Beta-Lactam Antibiotic Concentrations in Adipose, Bone, and Muscle Tissues	De Sutter PJ	Drug Metab Dispos	2023	10.1124/dmd.122.001129
21	Pharmacokinetics and Target Attainment of ss-lactam Antibiotics in Older People: A Systematic Review of Current Literature	Vervalcke J	Clin Pharmacokinet	2023	10.1007/s40262-022-01196-1
22	Verifying in vitro-determined enzyme contributions to cannabidiol clearance for exposure predictions in human through physiologically-based pharmacokinetic modeling	Yeung CHT	CPT Pharmacometrics Syst Pharmacol	2023	10.1002/psp4.12908
23	Foetal and neonatal exposure prediction and dosing evaluation for ampicillin using a physiologically-based pharmacokinetic modelling approach	Li S	Br J Clin Pharmacol	2023	10.1111/bcp.15589
24	Physiologically based pharmacokinetic model to predict drug concentrations of breast cancer resistance protein substrates in milk	Zhang T	Biopharm Drug Dispos	2022	10.1002/bdd.2335
25	Prediction of Tissue Exposures of Meropenem, Colistin, and Sulbactam in Pediatrics Using Physiologically Based Pharmacokinetic Modeling	Zhu S	Clin Pharmacokinet	2022	10.1007/s40262-022-01161-y
26	Population Pharmacokinetics of Vancomycin in Pregnant Women	Goyal RK	Front Pharmacol	2022	10.3389/fphar.2022.873439
27	Leveraging physiologically based pharmacokinetic modeling to optimize dosing for lopinavir/ritonavir with rifampin in pediatric patients	Salerno SN	Pharmacotherapy	2023	10.1002/phar.2703
28	Development and application of a pediatric mechanistic kidney model	Salem F	CPT Pharmacometrics Syst Pharmacol	2022	10.1002/psp4.12798
29	Physiologically Based Pharmacokinetic Modeling and Dose Adjustment of Teicoplanin in Pediatric Patients With Renal Impairment	Xu J	J Clin Pharmacol	2022	10.1002/jcph.2000
30	Optimal Teicoplanin Dosing Regimen in Neonates and Children Developed by Leveraging Real-World Clinical Information	Yamada T	Ther Drug Monit	2022	10.1097/FTD.0000000000000930
31	Development and Evaluation of a Virtual Population of Children with Obesity for Physiologically Based Pharmacokinetic Modeling	Gerhart JG	Clin Pharmacokinet	2022	10.1007/s40262-021-01072-4

32	Prediction of Moxifloxacin Concentrations in Tuberculosis Patient Populations by Physiologically Based Pharmacokinetic Modeling	Litjens CHC	J Clin Pharmacol	2022	10.1002/jcph.1972
33	Investigating the Critical Variables of Azithromycin Oral Absorption Using In Vitro Tests and PBPK Modeling	Guimarães M	J Pharm Sci	2021	10.1016/j.xphs.2021.09.013
34	Evidence-Based Guidelines for Drug Interaction Studies: Model-Informed Time Course of Intestinal and Hepatic CYP3A4 Inhibition by Clarithromycin	Kapetas AJ	AAPS J	2021	10.1208/s12248-021-00632-7
35	Ceftaroline Dosage Optimized for Pediatric Patients With Renal Impairment Using Physiologically Based Pharmacokinetic Modeling	Zhou J	J Clin Pharmacol	2021	10.1002/jcph.1944
36	Physiologically Based Pharmacokinetic Modeling for Selumetinib to Evaluate Drug-Drug Interactions and Pediatric Dose Regimens	Cohen-Rabbie S	J Clin Pharmacol	2021	10.1002/jcph.1935
37	Physiologically Based Pharmacokinetic Modeling of Meropenem in Preterm and Term Infants	Ganguly S	Clin Pharmacokinet	2021	10.1007/s40262-021-01046-6
38	Leveraging Physiologically Based Pharmacokinetic Modeling and Experimental Data to Guide Dosing Modification of CYP3A-Mediated Drug-Drug Interactions in the Pediatric Population	Salerno SN	Drug Metab Dispos	2021	10.1124/dmd.120.000318
39	Estimation of Ontogeny Functions for Renal Transporters Using a Combined Population Pharmacokinetic and Physiology-Based Pharmacokinetic Approach: Application to OAT1,3	Cristea S	AAPS J	2021	10.1208/s12248-021-00595-9
40	Application of Physiologically Based Pharmacokinetic-Pharmacodynamic Modeling in Preterm Neonates to Guide Gentamicin Dosing Decisions and Predict Antibacterial Effect	Neeli H	J Clin Pharmacol	2021	10.1002/jcph.1890
41	Next generation risk assessment (NGRA): Bridging in vitro points-of-departure to human safety assessment using physiologically-based kinetic (PBK) modelling - A case study of doxorubicin with dose metrics considerations	Li H	Toxicol In Vitro	2021	10.1016/j.tiv.2021.105171
42	Determination of vancomycin exposure target and individualised dosing recommendations for neonates: model-informed precision dosing	Tang Z	Int J Antimicrob Agents	2021	10.1016/j.ijantimicag.2021.106300
43	Dosage Adjustment for Ceftazidime in Pediatric Patients With Renal Impairment Using Physiologically Based Pharmacokinetic Modeling	Zhou J	J Pharm Sci	2021	10.1016/j.xphs.2021.02.001
44	Teicoplanin physiologically based pharmacokinetic modeling offers a quantitative assessment of a theoretical influence of serum albumin and renal function on its disposition	Emoto C	Eur J Clin Pharmacol	2021	10.1007/s00228-021-03098-w
45	Model-Informed Pediatric Dose Selection for Dapagliflozin by Incorporating Developmental Changes	Jo H	CPT Pharmacometrics Syst Pharmacol	2021	10.1002/psp4.12577
46	Physiologically based pharmacokinetic model of renally cleared antibacterial drugs in Chinese renal impairment patients	Cui C	Biopharm Drug Dispos	2021	10.1002/bdd.2258
47	Linezolid-induced thrombocytopenia in a child with a liver transplant: A case report	Lu X	Int J Clin Pharmacol Ther	2021	10.5414/CP203754
48	Sequestration of Voriconazole and Vancomycin Into Contemporary Extracorporeal Membrane Oxygenation Circuits: An in vitro Study	Raffaeli G	Front Pediatr	2020	10.3389/fped.2020.00468
49	Saliva versus Plasma Therapeutic Drug Monitoring of Gentamicin in Jordanian Preterm Infants. Development of a Physiologically-Based Pharmacokinetic (PBPK) Model and Validation of Class II Drugs of Salivary Excretion Classification System	Idkaidek N	Drug Res (Stuttg)	2020	10.1055/a-1233-3582
50	Integration of physiological changes during the postpartum period into a PBPK framework and prediction of amoxicillin disposition before and shortly after delivery	Dallmann A	J Pharmacokinet Pharmacodyn	2020	10.1007/s10928-020-09706-z
51	Formulation and Bioequivalence Testing of Fixed-Dose Combination Orally Disintegrating Tablets for the Treatment of Tuberculosis in the Paediatric Population	Dennison TJ	J Pharm Sci	2020	10.1016/j.xphs.2020.07.016
52	Physiologically based pharmacokinetic-pharmacodynamic evaluation of meropenem plus fosfomycin in paediatrics	Martins FS	Br J Clin Pharmacol	2021	10.1111/bcp.14456
53	Modelled plasma concentrations of pemafibrate with co-administered typical cytochrome P450 inhibitors clopidogrel, fluconazole or clarithromycin predicted by physiologically based pharmacokinetic modelling in virtual populations	Ogawa SI	Xenobiotica	2020	10.1080/00498254.2020.1793030
54	A Physiologically Based Pharmacokinetic Model of Ertapenem in Pediatric Patients With Renal Impairment	Ye L	J Pharm Sci	2020	10.1016/j.xphs.2020.06.010
55	First dose in neonates: pharmacokinetic bridging study from juvenile mice to neonates for drugs metabolized by CYP3A	Ye PP	Xenobiotica	2020	10.1080/00498254.2020.1768454
56	Use of normalized prediction distribution errors for assessing population physiologically-based pharmacokinetic model adequacy	Maharaj AR	J Pharmacokinet Pharmacodyn	2020	10.1007/s10928-020-09684-2
57	Drug Interactions for Low-Dose Inhaled Nemiralisib: A Case Study Integrating Modeling, In Vitro, and Clinical Investigations	Patel A	Drug Metab Dispos	2020	10.1124/dmd.119.089003
58	The development of a population physiologically based pharmacokinetic model for mycophenolic mofetil and mycophenolic acid in humans using data from plasma, saliva, and kidney tissue	Alsmadi MM	Biopharm Drug Dispos	2019	10.1002/bdd.2206
59	Preterm Physiologically Based Pharmacokinetic Model. Part II: Applications of the Model to Predict Drug Pharmacokinetics in the Preterm Population	Abduljalil K	Clin Pharmacokinet	2020	10.1007/s40262-019-00827-4
60	Using mechanistic physiologically-based pharmacokinetic models to assess prenatal drug exposure: Thalidomide versus efavirenz as case studies	Atoyebi SA	Eur J Pharm Sci	2019	10.1016/j.ejps.2019.105068
61	Application of Physiologically-Based and Population Pharmacokinetic Modeling for Dose Finding and Confirmation During the Pediatric Development of Moxifloxacin	Willmann S	CPT Pharmacometrics Syst Pharmacol	2019	10.1002/psp4.12446

62	Population Pharmacokinetic Modeling of Gentamicin in Pediatrics	Wang H	J Clin Pharmacol	2019	10.1002/jcph.1479
63	Development of a physiologically-based pharmacokinetic pediatric brain model for prediction of cerebrospinal fluid drug concentrations and the influence of meningitis	Verscheijden LFM	PLoS Comput Biol	2019	10.1371/journal.pcbi.1007117
64	Towards a Generic Tool for Prediction of Meropenem Systemic and Infection-Site Exposure: A Physiologically Based Pharmacokinetic Model for Adult Patients with Pneumonia	Thémans P	Drugs R D	2019	10.1007/s40268-019-0268-x
65	Clinical Pharmacokinetic Studies in Pregnant Women and the Relevance of Pharmacometric Tools	Dallmann A	Curr Pharm Des	2019	0.2174/1381612825666190320135137
66	Physiologically Based Pharmacokinetic Modeling for Trimethoprim and Sulfamethoxazole in Children	Thompson EJ	Clin Pharmacokinet	2019	10.1007/s40262-018-00733-1
67	Physiologically based pharmacokinetic modelling to predict exposure differences in healthy volunteers and subjects with renal impairment: Ceftazidime case study	Zhou L	Basic Clin Pharmacol Toxicol	2019	10.1111/bcpt.13209
68	Predicting Drug-Drug Interactions Between Rifampicin and Long-Acting Cabotegravir and Rilpivirine Using Physiologically Based Pharmacokinetic Modeling	Rajoli RKR	J Infect Dis	2019	10.1093/infdis/jiy726
69	Physiologically-Based Pharmacokinetic model for Ciprofloxacin in children with complicated Urinary Tract Infection	Balbas-Martinez V	Eur J Pharm Sci	2019	10.1016/j.ejps.2018.11.033
70	Intensified antibiotic treatment of tuberculosis meningitis	Cresswell FV	Expert Rev Clin Pharmacol	2019	10.1080/17512433.2019.1552831
71	Optimizing Antibiotic Drug Therapy in Pediatrics: Current State and Future Needs	Le J	J Clin Pharmacol	2019	10.1002/jcph.1128
72	Development of a physiologically based pharmacokinetic model for mefloquine and its application alongside a clinical effectiveness model to select an optimal dose for prevention of malaria in young Caucasian children	Johnson TN	Br J Clin Pharmacol	2019	10.1111/bcp.13764
73	Treatment and outcomes in children with multidrug-resistant tuberculosis: A systematic review and individual patient data meta-analysis	Harausz EP	PLoS Med	2018	10.1371/journal.pmed.1002591
74	Modelling the long-acting administration of anti-tuberculosis agents using PBPK: a proof of concept study	Rajoli RKR	Int J Tuberc Lung Dis	2018	10.5588/ijtld.17.0515
75	Development of a Physiologically Based Pharmacokinetic Model of Ethionamide in the Pediatric Population by Integrating Flavin-Containing Monooxygenase 3 Maturational Changes Over Time	Nguyen PTT	J Clin Pharmacol	2018	10.1002/jcph.1133
76	A Physiologically-Based Pharmacokinetic Model to Describe Ciprofloxacin Pharmacokinetics Over the Entire Span of Life	Schlender JF	Clin Pharmacokinet	2018	10.1007/s40262-018-0661-6
77	Using a Vancomycin PBPK Model in Special Populations to Elucidate Case-Based Clinical PK Observations	Emoto C	PT Pharmacometrics Syst Pharmacol	2018	10.1002/psp4.12279
78	Pharmacokinetic/pharmacodynamic drug-drug interactions of avatrombopag when coadministered with dual or selective CYP2C9 and CYP3A interacting drugs	Nomoto M	Br J Clin Pharmacol	2018	10.1111/bcp.13517
79	Development of Guanfacine Extended-Release Dosing Strategies in Children and Adolescents with ADHD Using a Physiologically Based Pharmacokinetic Model to Predict Drug-Drug Interactions with Moderate CYP3A4 Inhibitors or Inducers	Li A	Paediatr Drugs	2018	10.1007/s40272-017-0270-0
80	Development of an Adult Physiologically Based Pharmacokinetic Model of Solithromycin in Plasma and Epithelial Lining Fluid	Salerno SN	CPT Pharmacometrics Syst Pharmacol	2017	10.1002/psp4.12252
81	Development of a paediatric physiologically based pharmacokinetic model to assess the impact of drug-drug interactions in tuberculosis co-infected malaria subjects: A case study with artemether-lumefantrine and the CYP3A4-inducer rifampicin	Olafuyi O	Eur J Pharm Sci	2017	10.1016/j.ejps.2017.05.043
82	Pharmacokinetic changes of norfloxacin based on expression of MRP2 after acute exposure to high altitude at 4300m	Luo B	Biomed Pharmacother	2017	10.1016/j.biopha.2017.02.092
83	Development of a Pediatric Physiologically-Based Pharmacokinetic Model of Clindamycin Using Opportunistic Pharmacokinetic Data	Hornik CP	Clin Pharmacokinet	2017	10.1007/s40262-017-0525-5
84	CaCO3 nanoparticles as an ultra-sensitive tumor-pH-responsive nanoplatform enabling real-time drug release monitoring and cancer combination therapy	Dong Z	Biomaterials	2016	10.1016/j.biomaterials.2016.09.025
85	The amikacin research program: a stepwise approach to validate dosing regimens in neonates	Smits A	Expert Opin Drug Metab Toxicol	2017	10.1080/17425255.2017.1234606
86	Physiologically Based Pharmacokinetic Prediction of Linezolid and Emtricitabine in Neonates and Infants	Duan P	Clin Pharmacokinet	2017	10.1007/s40262-016-0445-9
87	Clinical research in neonates and infants: Challenges and perspectives	Coppini R	Pharmacol Res	2016	10.1016/j.phrs.2016.04.025
88	Development of a physiologically based pharmacokinetic model of actinomycin D in children with cancer	Walsh C	Br J Clin Pharmacol	2016	10.1111/bcp.12878
89	A physiologically based pharmacokinetic modeling approach to predict drug-drug interactions between domperidone and inhibitors of CYP3A4	Templeton I	Biopharm Drug Dispos	2016	10.1002/bdd.1992
