# Supplementary material for: Physiologically Based Pharmacokinetic Modeling of Antibiotics in Children: Perspectives on Model-Informed Precision Dosing
Source: Antibiotics (Basel). 2025 May 24;14(6):541. doi: 10.3390/antibiotics14060541 (PMC12189772; doi:10.3390/antibiotics14060541)
Supplement: Supplementary file 1 [file antibiotics-14-00541-s001.zip › Table S3_List of antibiotics PBPK model.pdf]

Table S2 List of antibiotics PBPK model

Number	Drug	Validation tissue	Administration route	Verified PK parameters	Full or minimal in case of using Simcyp	Pediatric age population	Objective (in addition to age-dependent optimal dose)	Target infection	Assumed target bacteria	Target PK/PD indices	Adult model verification	Software	PMID	Journal	Year
1	Polymyxin-B<br>Amikacin<br>sulbactam	Plasma<br>Heart<br>Lung<br>Skin	IV	AUC, C_{max}	-	2-<18 years	Tissue target attainment for acinetobacter. Baumannii	-	*Acinetobacter baumannii*	Polymyxin B: $fAUC_{0–24}$/MIC>=8.2<br>Amikacin: $fAUC_{0–24}$/MIC>=80<br>Sulbactam: fT>MIC>=40%	This study	PK-Sim v10.0	39435440	Front Microbiol	2024
2	Cefotaxime	Plasma	IV	$AUC_{0\text{-}inf}$, C_{max}	-	Neonate	Dose selection for preterm and term neonates	-	Enterobacteriaceae (PNA: 0-6 days)<br>*Staphylococcus Aureus* (PNA:7-28 days)	fT>MIC=100%	This study	PK-Sim v11.0	38460573	J Pharm Sci	2024
3	Amikacin<br>Fosfomycin	Plasma	IV	AUC, C_{max}	Full	Neonate	Dose selection for neonate	Sepsis	*Enterobacterales*	Amikacin: C_{max}/MIC>10, AUC/MIC>23 for stasis and >82 for 1-log_{10} CFU/mL reduction<br>Fosfomycin: AUC/MIC>21.4 for stasis and >62.5 for 1-log_{10} CFU/mL reduction	This study	Simcyp v22.0	38108548	CPT Pharmacometrics Syst Pharmacol	2024
4	Linezolid	Plasma<br>Cranial CSF	IV	AUC	Full	0.25-21 years	Prediction of cranial cerebrospinal fluid profile	Tuberculous meningitis	*Mycobacterium tuberculosis*	AUC/MIC>119<br>T>MIC>80%	This study	Simcyp v19.0	37107064	Antibiotics (Basel)	2023
5	Azithromycin	Plasma	IV, PO	AUC, C_{max}, T_{max}	-	0.5-2, 3-6, 7-12, and 13-18 years	Dose selection for children of different ages	Community-acquired pneumonia	-	fAUC/MIC>=25	This study	PK-Sim v8.0	37080927	Biopharm Drug Dispos	2023
6	Daptomycin<br>Ceftaroline	Plasma<br>Lung<br>Skin	IV	AUC, C_{max}	Full	2-<6, 6-<12, and 12-<18 years	Dose selection in paediatrics, renally impaired, obese and geriatrics	Bacteraemia	MRSA	Daptomycin: fAUC\MIC>40<br>Ceftaroline: fT>MIC>=35%	This study	PK-Sim v9.0	37005335	Br J Clin Pharmacol	2023
7	Ampicillin	Plasma	IV, PO	$AUC_{0\text{-}inf}$, C_{max}	Full	Neonate	Dose selection for neonate and during the intrapartum period	Neonate: Sepsis<br>Intrapartum pediod: prophylaxis for Group B Streptococcus infection	*Listeria monocytogenes* , *Escherichia coli* , Group B *Streptococcus*	fT>MIC>=50%, >=75%, and >=100%	This study	Simcyp v21.0	36357171	Br J Clin Pharmacol	2023
8	Meropenem<br>Sulbactam<br>Colistin	Plasma<br>Heart<br>Lung<br>Skin<br>Brain ISF (colistin)	IV	$AUC_{0\text{-}24h}$, C_{max}	-	2-<18 years	Tissue target attainment for acinetobacter. baumannii with and without renal impairment (CrCL<50 mL/min)	Lung, skin, heart-related infections	*Acinetobacter baumannii*	Meropenem: fT>MIC>=40%<br>Sulbactam: fT>MIC>=60%<br>Colistin: fAUC/MIC>=7.4	This study	PK-Sim v10.0	35947360	Clin Pharmacokinet	2022
9	Teicoplanin	Plasma	IV	$AUC_{0\text{-}t}$, C_{max}	-	2-12 years	Dose selection in pediatrics with renal impairment (GFR: 60-89, 30-59, 15-29 mL/min/1.73m2)	-	*Staphylococcus aureus*	fAUC/MIC>=345	This study	GastroPlus v9.8	34761398	J Clin Pharmacol	2022
10	Clindamycin<br>Trimethoprim/sulfamethoxazole	Plasma	IV, PO	AUC_{ss}, C_{max}	-	Clindamycin: >2-6 and >6-18 years<br>Trimethoprim/sulfamethoxazole: >2-12 and >12-18 years	Dose selection in pediatrics with obese	-	-	-	Previous study	PK-Sim v9.0	34617262	Clin Pharmacokinet	2022
11	Moxifloxacin	Plasma	IV, PO	$AUC_{0\text{-}24h}$, C_{max}	Full	3 months-<2, 2-<6, and 6-14 years	Effect of DDI with rifanpicin on PK	Tuberculous infection	Multidrug-resistance tuberculosis	-	This study	Simcyp v19.0	34554580	J Clin Pharmacol	2022
12	Azithromycin	Plasma	IV, PO	AUC, C_{max}, T_{max}	Minimal	0.5-2, >2-6, >6-12, and >12-16 years	Identification of critical variables affecting oral absorption	-	-	-	This study	Simcyp v18.2	34530004	J Pharm Sci	2021
13	Ceftaroline	Plasma	IV	$AUC_{0\text{-}t}$, C_{max}, $t_{1/2}$, V_{ss}, CL	-	Neonate with full-term and preterm, and 1 and 6 years	Dose selection in pediatric patients with renal impairment (GFR: 60-89, 30-59, 15-29, <15 mL/min/1.73m2)	Acute bacterial skin and skin structure infections and community-acquired bacterial pneumonia	*Staphylococcus aureus* , *Streptococcus pneumoniae*	fT>MIC>=36% for *Staphylococcus aureus*<br>fT>MIC>=44% for *Streptococcus pneumoniae*	This study	GastroPlus v9.7	34329494	J Clin Pharmacol	2021
14	Meropenem	Plasma	IV	$AUC_{0\text{-}inf}$, CL	-	<3 month	Dose selection for preterm and term infants	Complicated intra-abdominal infection	-	fT>MIC>=50% for a MIC of 4 mg/L<br>fT>MIC>=75% for a MIC of 2 mg/L	This study	PK-Sim v8.0	34155614	Clin Pharmacokinet	2021
15	Solithromycin	Plasma	IV, PO (capsule and suspension)	$AUC_{0\text{-}inf}$, $AUC_{0\text{-}t}$, C_{max}, CL	-	1-<6 months, 0.5-<2, 2-<6, 6-<12, and 12-<18 years	Effect of DDI with midazolam or ketoconazole on PK	-	-	-	This study	PK-Sim v9.0	34154994	Drug Metab Dispos	2021
16	Gentamicin	Plasma	IV	AUC, C_{max}	Minimal	Neonates with PNA of 0-7 or 8-28 days and PMA of <30, 30-34, or >34 weeks	Dose selection in preterm neonate based on developed PBPK-PD model	-	*Escherichia coli*	C_{max}/MIC>=8<br>Trough level<=1 µg/mL	This study	Simcyp v20.0	33945155	J Clin Pharmacol	2021
17	Ceftazidime	Plasma	IV	$AUC_{0\text{-}t}$, C_{max}	-	1 month to 12 years	Dose selection in pediatric patients with renal impairment (GFR: 60-89, 30-59, 15-29, <15 mL/min/1.73m2)	-	-	fT>MIC>=70%	This study	GastroPlus v9.7	33556385	J Pharm Sci	2021
18	Gentamicin	Plasma<br>Saliva	IV	-	Full	Premature infants with a median PNA of 0.08 days	TDM in saliva in premature infants	-	-	-	This study	PK-Sim v8.0	32877949	Drug Res (Stuttg)	2020
19	Fosfomycin<br>Meropenem	Plasma	IV, PO (fosfomycin)	AUC, C_{max}	-	0.1-12 years	Evaluation of joint probability of target attainment in multidrug-resistant infection	-	*Enterobacteriaceae* , *Pseudomonas aeruginosa*	Fosfomycin: fAUC/MIC>=40.8<br>Meropenem: fT>MIC>=40%	This study	PK-Sim v8.0	32638408	Br J Clin Pharmacol	2021
20	Ertapenem	Plasma	IV	$AUC_{0\text{-}t}$, C_{max}	-	0-12 years	Dose selection in pediatric patients with renal impairment (GFR: 60-89, 30-59, 15-29, <15 mL/min/1.73m2)	-	-	fT>MIC>=40%	This study	GastroPlus v9.7	32565352	J Pharm Sci	2020
21	Moxifloxacin	Plasma	IV, PO	$AUC_{0\text{-}24h}$, C_{max}	-	3 months-<2, 2-<6, and 6-<12, 12-<18 years	Dose selection in pediatric patients (model verification with the popPK model)	-	-	AUC>=45 mg*h/L<br>Cmax=2~6 mg/L	Previous study	PK-Sim v4.2	31310051	CPT Pharmacometrics Syst Pharmacol	2019
22	Trimethoprim/sulfamethoxazole	Plasma<br>Urine	IV, PO	AUC, C_{max}	-	2-5 months, 5 months-1 year, >1-6, >6-12, >12-18 years	Dose selection in pediatric patients	-	MRSA	Trimethoprim: 20.6<AUC_{ss}<141.8 mg*h/L, C_{max}<13.6 mg/L<br>Sulfamethoxazole: AUC_{ss}<4119.4 mg*h/L, C_{max}<372 mg/L	This study	PK-Sim v5.5	30840200	Clin Pharmacokinet	2019
23	Ciprofloxacin	Plasma<br>Urine<br>Saliva	IV, PO	-	-	3, 6 and 18 months and 4, 8 and 12 years	Prediction of plasma and urine profiles in pediatric patients with complicated urinary tract infection	Complicated urinary tract infection	-	-	This study	PK-Sim v7.2.1	30503378	Eur J Pharm Sci	2019
24	Ethionamide	Plasma	PO	AUC, C_{max}, T_{max}, CL	Full	0 months- 2 years, 2-6 years, 6-12 years	Verification of the effect of FMO3 maturational changes on the exposure with increasing age in children	Tuberculous infection	Drug-resistance tuberculosis	-	This study	Simcyp v15.0	29878384	J Clin Pharmacol	2018
25	Vancomycin	Plasma	IV	AUC, C_{max}	Full	2.6 days-7 years 7 months	Changes in the PK profiles in special populations	-	-	-	This study	Simcyp v16.0	29446256	CPT Pharmacometrics Syst Pharmacol	2018

**26**	Clindamycin	Plasma<br>Urine	IV	-	-	0-5 months, >5 months-1 year, >1-6, >6-12, and >12-18 years	Dose selection for children of different ages	Community-acquired MRSA infection	MRSA	-	This study	PK-Sim v5.5	28290120	Clin Pharmacokinet	2017
**27**	Linezolid<br>Emtricitabine	Plasma	IV, PO	AUC, C_{max}	Linezolid: minimal<br>Emtricitabine: full	Neonate (1-28 days) and 3-12 months, 1-2, 3-6, 7-12, and 13-17 years	PK understanding in neonates and infants	-	-	-	This study	Simcyp v14.1	27596256	Clin Pharmacokinet	2017
